# Should we change targets and methods of early intervention in autism, in favor of a strengths-based education?

**DOI:** 10.1007/s00787-017-0955-5

**Published:** 2017-02-08

**Authors:** Laurent Mottron

**Affiliations:** University of Montréal, Montréal, Québec Canada

**Keywords:** Autism, Intelligence, Early intensive behavioral intervention, Autistic strengths

## Abstract

Early intensive behavioral intervention (EIBI) and its recent variant, naturalist developmental behavioral intervention (NDBI) aim to increase socialization and communication, and to decrease repetitive and challenging behaviors in preschool age autistic children. These behaviorist techniques are based on the precocity and intensity of the intervention, face-to-face interaction, errorless learning, and information fragmentation. Once considered to be “scientifically proven”, the efficacy of these approaches has been called into question in the last decade due to poor-quality data, small effects, low cost-efficiency, and the evolution of ethical and societal standards. Grounded on a reappraisal of the genetic and cognitive neuroscience of autism, we question three aspects of EIBI/NDBI: their focus on prerequisites for typical socio-communicative behaviors, their lack of consideration of autistic language development and learning modes, and their negative view of repetitive behaviors and restricted interests. We propose alternative predictions for empirical validation, based on the strengths of prototypical autistic children: (a) their non-verbal intelligence should be normally distributed and within the normal range; (b) improving access to non-communicative verbal and written auditory language material should favor their subsequent speech development and (c) decrease their problematic behavior; (d) lateral tutorship should increase the well-being of children and parents to a greater extent than personalized, face-to-face interventions by professionals; (e) admission to regular, but supervised daycare centers, combined with parental support and on-site crisis solving, represents a more cost-effective educational intervention than EIBI/NDBI.

## A brief history of early, intensive behavioral intervention for autistic preschoolers: where are we now?

In developed countries, autism can be diagnosed at approximately 2 years of age, making it possible to intervene before the child starts school. The interventions employed worldwide are grouped under the umbrella term “early, intensive behavioral intervention” (EIBI) in meta-analyses and practice guidelines for psychosocial interventions targeting the “core autism feature of impaired reciprocal social communication and interaction” [47]. The learning models, techniques, and targets of EIBI have been strongly influenced throughout its existence by methods inspired from studies of animal behavior. The interventions developed, initially known as applied behavioral analysis or ABA [36], were also delivered professionally and face-to-face. Following the landmark study by [35], the publication of short-term empirical measures of the effect of intervention on IQ, autistic signs, and adaptation led to the integration of ABA into evidence-based practices, as well as the cliché of ABA being the only “scientifically proven” intervention (see [21] for an opinion).

Several aspects of ABA intervention have evolved. Naturalistic developmental behavioral interventions (NDBI), which are peer-mediated, community-based interventions delivered by a child’s parents in natural settings, address the lack of generalization and the laboratory style of initial ABA interventions. They are still largely faithful to operant conditioning learning procedures, whereas some have distanced themselves from it (see [31, 48] for a review). Another recent approach, the Denver model [12, 57], aims to combine ABA with knowledge about the developmental psychology of the *typical* child. This approach has led to a shift in the focus of ABA techniques and targets, from the learning of autonomy through conditioning, to the enrichment of the prerequisites of typical social communication routines (joint attention, imitation) through a combination of play and conditioning procedures.

An apparent consensus has emerged over the last 40 years concerning the principles, targets, and efficacy measures of EIBI [26], with only a few exceptions (e.g., [59]). Despite their differences and apparent or claimed evolution, the various types of EIBI “are firmly grounded in principle and the science of learning and (…) fully meet criteria as ABA techniques” [60]. Therefore, the various currents of EIBI display a considerable overlap in terms of the techniques used and the underlying principles: *precocity* (the earlier the intervention is applied, the more effective it is likely to be), *intensity* (more intensive interventions are preferred, with as many as 25 h/week frequently recommended), highly structured and constantly monitored interventions, *normativity* (the less autistic the child is at the end of the intervention, the more successful the intervention is considered to have been), task-decomposition (information to be learned is broken down), the use of external or natural reinforcement, and extinction procedures. Intervention targets map the definition of a combination of negative socio-communicative symptoms (“lack” or limitation of socially oriented behaviors) and positive, non-social symptoms (an “excess” of restricted interests and repetitive behaviors). EIBI aims to render this bumpy profile flat and less autistic: increase prerequisites of typical socially oriented behaviors and communication, while suppressing repetitive behaviors and autistic interests. EIBI has been recommended worldwide, and most national guidelines for autism even specify the amount of therapy (e.g., USA: [64, 65, 68]; France: [25]; Quebec: [27]), often as a means of opposing non-scientific interventions, with the remarkable exception of the UK.

However, the early twenty-first century has seen the emergence of critics of EIBI. An influential autism research expert [13] questioned the presuppositions and ethical issues related to EIBI, and criticized the use of low standards in autism intervention research. Its underlying principles and targets, the empirical demonstration of its efficacy, its cost-efficiency, and ethical justification are now widely questioned ([22]; see [40] for a review). Careful studies of the variables used to assess the effects of intervention have highlighted their inadequacy for detecting changes [38]. In the UK, a series of studies has emerged on communication-based, parental guidance interventions (e.g., PACT; [1]). In striking contrast to both the ABA and the Denver model, these interventions are based on low-intensity parental guidance while retaining the same underlying assumptions as ABA interventions, except for intensity. They have considerably improved the scientific standards of intervention studies. This line of research led to the first large-scale (*N* = 152) randomized controlled trial on early intervention [23], showing weak, but demonstrable, effects on autistic symptoms 6 years after the intervention [52].

Meta-analyses of intervention studies (see [55] for an example) continue to perpetuate the consensual view that EIBI is effective, whereas UK experts have produced a practice guide [47] that assesses the strength of proof for all available early intervention studies. NICE practice guides are based on standards common to other health disciplines. Their quality standards are, therefore, substantially higher than those of other autism practice guides. The NICE guide highlights the limited nature of the changes that can be unambiguously attributed to the intervention itself. One of the major conclusions of this guide is that the quality of intervention research has been low or very low, and that it is therefore not possible to recommend ABA, or, more generally, EIBI. This conclusion contrasts strongly with those put forward in the US, French, and Canadian recommendations.

Our aim here was to question EIBI from another angle, that of its targets and principles. We will discuss the following issues: the legitimacy of targeting the prerequisites of learning or typical social functioning; whether the timing of the intervention and the techniques used improve the way autistic children learn language; and whether autistic repetitive behavior and restricted interests can be used as cognitive strengths, rather than as a motivator or suppressed as disturbing behaviors. We will then propose a series of alternative intervention principles, with the aim of inspiring a new generation of clinical trials.

Given the heterogeneity of individuals diagnosed with autism, according to current definitions, we will limit our discussion to the core autistic phenotype: idiopathic (or primary, or non-syndromic) autistic preschool children with strong, unambiguous and reliable autistic phenotypes, in general functionally non-verbal, referred to here as “prototypical” autism. Our discussion and predictions do not, therefore, necessarily apply to early verbal or mild autistic phenotypes, or, at the other end of the spectrum, to “syndromic” autism accompanied by an identifiable neurodevelopmental condition and unquestionable non-verbal intellectual disability.

## A dubious intervention target: prerequisites of learning and typical social functioning

Despite supposed differences in techniques and underlying philosophy, the two principal forms of EIBI, ABA and the Denver model, both target the *prerequisites* of the abilities they want to help the child master [36, 57]. This notion of a “prerequisite” implies that the typical, non-autistic early manifestations of the targeted function must be mastered before the autistic child can develop more complex, or mature non-autistic behaviors. For example, looking into the eyes of the person with whom the child is interacting is considered to be a prerequisite for his future abilities in reciprocal social interaction, or for language and learning in general. Interventions therefore focus on training the autistic child to do this.

In the ABA approach, the notion of “prerequisites” is summed up in the “learn to learn” principle [36]. In practical terms, before the ABA technique can be used for vocabulary training, for example, an autistic toddler must be trained to accept the format of the personalized, face-to-face training sessions through which ABA training is achieved. This intervention sequence is justified by the argument that autistic children would not learn by themselves, because they lack the innate ability and disposition to learn spontaneously, which must therefore be constructed artificially. This involves training the child to look at the teacher, to remain seated on a chair, face-to-face with the teacher, and to stop making repetitive movements or spontaneously focusing on a preferred object of interest, all of which is considered to be essential for the child to be able to benefit from what he or she is learning. The same rationale applies to the ABA interventions themselves, which take the form of graduated steps, from the simplest to the most complex. Each step must be performed without error before the next teaching target is attempted.

The main objection to Lovaas “learn to learn” principle is that speech, motor skills, and many cognitive abilities are not—even typically—learned through directed, hierarchical, reinforced training sessions. Behaviorism is a restricted, simplified model of typical behavior that does not consider either genetic constraints on developmental pathways or non-contingent, unfragmented, non-hierarchical, non-error-free forms of learning. The behaviorist model misses a dozen other learning mechanisms, particularly implicit learning. The assumption that autistic children do not learn by themselves is clearly untrue. Implicit learning has repeatedly been demonstrated in autistic individuals [7, 46] and is actually slowed down by explicit instructions [29]. Autistic children, and not only savants, can spontaneously learn large amounts of complex information. The immediate value of the information they choose to learn may not be obvious, but autistic children resist conventional ways of learning, precisely because they learn by themselves, rather than because they are “incapable of learning” (see Dawson et al., 2008 for an informed discussion). However, behaviorists claim to have elucidated learning rules that apply across a continuum extending from young mice to old men. Proponents of this school of thought are not ready to accept the possibility that learning constraints may be species-specific, or differ between neurodevelopmental variants, to such a point that the very principles of behaviorism may be undermined.

In the Denver model targets, the alleged “prerequisites” for the subsequent mastering of socio-communicative abilities are joint attention, shared emotion, pointing, and face-to-face imitation. The justification of this intervention strategy is that these behaviors *precede* more complex socio-communicative behaviors in typical children. Their presence and magnitude may be *predictive* of the subsequent development of socio-communicative abilities. The Denver program involves intensive training in these behaviors, with the aim of “kick-starting” an underdeveloped function that may, through “brain plasticity” and intensive training, catch up to levels similar to those of typical children.

The assumption that not reaching a developmental milestone at the age at which it is typically attained precludes the further development of a given function may, however, not be valid if the trainee follows a specific developmental route which does not cross typical prerequisites. While this notion of prerequisites applies correctly to the ordered sequence of certain species-specific behaviors, its generalization to education is limited by the existence of several different, possibly incompatible developmental routes to the same milestone, even in typical individuals. Some steps may be delayed, missing, or their order reversed. For example, not all children crawl before walking [24], and typical children follow diverse routes to reading [10].

A related objection to the “prerequisite” rationale used to favor routine social targets in the Denver model is that typical socio-communication prerequisites are *indices* of a function, rather than the function itself. For example, smiling may only loosely reflect altruistic intentions, whereas, conversely, intense socialization is possible without smiling. Reduced socio-communicative behaviors are a key element in the early diagnosis of prototypical autism, but it remains unclear to what extent such behavior reflects actual peer bonding, as autistic toddlers have been shown to display typical attachment. A large range of social variables reflecting social abilities are actually normal when empirically measured in isolation in autistic adults. This finding is potentially disappointing for social cognitive neuroscientists trying to identify endophenotypes, but it may be good news for autistic individuals. Indeed, there is a major discrepancy between the oddity of autistic social behaviors, clinically measurable through standardized diagnostic instruments, the brain correlates of social competence in autistic individuals [50], and the actual ability of these individuals to perform tasks by assessing key components of human social bonding. Such discrepancies have been reported, for example, for mimetic desire [20], social orientation [45], social facilitation [30], and the automatic facial mimicry response [61]. These negative findings suggest that a clear distinction should be made between an abstract function of socialization, common to all members of the human species, and its visible manifestations, which may vary between subgroups of humans. It may, therefore, be inappropriate to evaluate socialization through their visible manifestations. Conversely, evaluations of socialization should focus on actual integration into the ordinary world rather than on indices and predictors of these abilities in non-autistic subjects. Socialization is a legitimate target, but its abstraction is required. Indices of socialization in autistic people are so different from those in non-autistic individuals that we may be missing the point by concentrating on non-autistic repertoires of socio-communicative behaviors.

The external validity of the “prerequisite” framework is even more limited if extended to encompass developmental variants, such as autism. Approaches targeting the prerequisites for attainment of a typical function assume that autistic children can become competent in the function concerned only if they follow the same steps as typical children, because these steps are simply *delayed*. We favor the view that autistic people follow a different pathway through the developmental sequences that result in maturation and social and language acquisition. Concerning early manifestations of peer relations, the “negative symptoms” of autistic individuals in the social domain may reflect the replacement of these manifestations by others within the developmental sequence of acquiring social competence. For example, reduced spontaneous *immediate* face-to-face imitation may be replaced by *delayed* imitation, without a detrimental influence on long-term competence. In the domain of communication, discussed below, the delay in speech development may reflect a greater interest in written material, supplanting efforts to speak, and an absence of overt social overtures between the ages of 2 and 5 years may reflect the use of non-social and non-communicative routes as the principal mans of language learning.

Finally, studies indicating, for example, that good pointing behavior during infancy is predictive of good verbal abilities later in life [43, 63] may simply be showing that the people farthest from an autistic phenotype during infancy are also those farthest from an autistic phenotype in adulthood. It does not necessarily mean that improving the pointing ability of *a specific autistic child* results in more typical socio-communicative abilities in adulthood. Metaphorically, more puppies than kittens swim, but training a cat to swim at a young age will not make that cat any more like a dog as an adult. Moreover, the late mastering of speech in strong autistic phenotypes [51], which, by definition, lack typical socio-communicative indices, demonstrates the absence of a direct relationship between these indices and the level of speech attained by the time an individual reaches adulthood. No valid information is available concerning the adaptive value of the EIBI strategy for the well-being of autistic adults. The focus on “prerequisites” of typical social function, even if relevant for typical children, cannot be used to prioritize socially oriented behavioral targets. We propose, therefore, to focus parent–child and caretaker–child interactions on the enrichment of and access to the child’s non-social interests as a goal of intervention, in place of the focus on the enrichment of typical socio-communicative prerequisites. Focussing on actual exchange, however, atypical its form may appear, follows Vigotsky’s “zone of proximal development” [18] while allowing, even belatedly, autistic children to converge towards more typical socio-communicative routines.

## Would it not be better to work with the autistic pathway for language development, rather than against it?

Language development in autism is widely regarded to be extremely variable, to the point that dramatic differences in speech onset has determined DSM IV sub-grouping of the autism spectrum. Language abilities are now one of the “clinical specifiers” of an autism diagnosis in the DSM-5. However, this variability can be structured by a few prototypical pathways within the autism spectrum. In prototypical autism, with which we are concerned here, the developmental sequence of autistic speech follows a “bayonet”-shaped pathway ([34]; see [40] for a discussion and [5] for a review). After the unremarkable acquisition of a handful of words at approximately 18 months of age, speech development appears to halt, reaching a plateau, or even regressing. Some children present echolalia during this period. Most of them just do not talk, whereas they may understand some instructions and written labels. This interruption of expected speech development coincides with the apparition of atypical socio-communicative behaviors. In most cases [67], this non-verbal plateau is followed by relatively rapid, but atypical, speech development at the age of about 40–60 months, including immediate and delayed echolalia and pronoun reversals (“you” for “I”). These atypical features are transient, usually persisting for less than 2 years, and are gradually replaced by functional spoken language. Although functional speech is the most common outcome, speech levels at school age and during adulthood range from the total absence of speech, unresponsiveness to verbal overtures or questions, idiosyncratic semantic associations, and an apparent lack of speech comprehension to functional or fluent speech for most autistic people [2].

Autistic speech development represents an altered developmental sequence, in which the apparently missing steps may be unnecessary, or manifest an inverse order of learning relative to that of typical preschoolers. First, intervention at this age has no significant, demonstrated influence on further speech development [47]. Second, speech levels and autistic signs between the ages of 2 and 4 years are not predictive of the levels of intelligence and adaptation subsequently attained. Individuals with strong autistic phenotypes and normal non-verbal intelligence generally end up speaking, even if they pass through unconventional steps. Non-verbal intelligence is predictive of future speech levels, whereas repetitive behaviors are not [67]. Third, attempts to drive autistic preschool children into typical socio-communicative routines is the least efficient and the most time-consuming way to provide them with information relevant for language acquisition. Autistic children are initially more focused on non-communicative speech, but use it for communicative purposes [53]. A substantial portion of them prefer written material over oral communication at this age. The detection and recognition of letters and numbers, and sometimes of complex written material, frequently occurs before the mastery of speech in autistic children [19, 32, 66]. Indeed, absent or atypical communicative oral language is commonly associated with hyperlexia, an early knowledge of written codes and intense interest in letters, which appears at the age of 2–3 years [45]. There are documented examples that hyperlexia leads to decoding abilities about 3 years ahead of those in typical children (see [49] for a review). This development of reading before oral speech is another indication that children with prototypical autism do not follow the typical developmental sequence of babbling and communicative one and two word sentences, followed by speech, and then reading.

In summary, the reduced sequence of communicative overtures—delayed echolalia—enhanced decoding abilities—speech delay-late attainment of functional speech, suggests that *autistic children mostly learn language in a non*-*communicative manner*. This counterintuitive idea has major consequences for interventions targeting autistic preschool children. The normocentric, step-by-step, prerequisite approach of classical ABA to the prosthetic construction of language should be abandoned. The exclusive emphasis on language prerequisites in the hopes of favoring further speech of the Denver model and NDBI, and even the focus of PACT on communication synchrony, may miss the specific autistic orientation towards and facility for non-social language. This suggests that it may be more constructive to complement the natural communication routine with more exposure to non-social oral language and written linguistic structures, rather than trying to modify what may be a non-modifiable constraint. To use an analogy, one should still talk to deaf children, as they may still grasp lip movements and facial cues, and benefit from an interaction which is typical from the parent’s point of view. However, if one wants to transmit sufficiently complex information, it is better to add sign language, even if it seems distant from typical communication.

## Reconsideration of our attitude towards early repetitive behaviors

In addition to its socio-communicative aspects, autism is also characterized by “restricted interest and repetitive behaviors” (RIRBs). Some historical details are relevant here. In early editions of the DSM, RIRBs accounted for only one-third of the possible behaviors used to diagnose autism. Research on RIRBs lagged far behind that of the social, communicative, and imaginative aspects of autism. The overriding view of RIRBs in the 1990s was that they were apparently nonspecific and equally likely to be found in autism and various other intellectual disabilities. However, another line of research initiated in the UK by U. Frith and K. Plaisted, and in Canada by our group, focused on the intrinsic link between these behaviors and autism. The research focused on the possible association between RIRBs, such as intense interests and autistic peaks of ability, with cognitive strengths and atypical features of information processing, related, in particular, to perception. We developed the “enhanced perceptual functioning” (EPF) model to account for unique aspects of autistic perception. The EPF model highlights the role, performance, and autonomy of perception in autistic cognition, and its contribution to autistic expertise, our non-offensive term for autistic savant abilities. One of the main proposals emanating from this model is that perception influences overt behavior and contributes to intelligence to a much greater extent in autistic than non-autistic individuals. Some RIRBs (e.g., an early interest in letters, staring at objects for longer) are, thus, manifestations of sophisticated information processing, as concluded from their correlation with perceptual cognitive peaks in adults [8]. The heterogeneity of RIRBs became evident in multiple large-scale studies attempting to group these behaviors into more homogeneous subgroups. It was found that the degree of specificity to autism differed between RIRBs, with some behaviors, such as lateral glances and longer visual inspection, being more specific to autism than others. It is now accepted that RIRBs are heterogeneous, involving various mechanisms with contrasting relationships to intelligence [3], but an intrinsic relationship to autism. In the latest edition of the DSM, RIRBs account for more than half of the possible manifestations of autism. Importantly, perception-related behaviors are now included among RIRBs, although unfortunately as “sensory” behaviors, a term that entirely ignores their relationship to intelligence and motivation.

Interventions have not been updated to take this new school of thought into account. There is no evidence to suggest that it is either possible [6] or beneficial [15] to globally reduce repetitive behaviors. The way in which most RIRBs continue to be seen in intervention programs is exemplified by their grouping with “problematic” behaviors in intervention studies, even being viewed as a justification for punishment [37]. Autistic interests are at best (NDBI) being used to increase engagement between the child and their parent/therapist as an opportunity to make requests or as positive reinforcement of social communication routines. Even the most recent adaptation of NDBI (see [60] for a review) has amongst its aims “controlling access to material of interest, playful obstruction, expectant waiting, violating a routine, using material that require assistance” ([60] pp 2419). Their behavioral manifestations are neither used nor, obviously, favored or enriched. Despite the relationship between RIRBs and positive emotions [42], they are still seen in a negative light and are assimilated with egodystonic thoughts or actions in the wording of the DSM-V. The Denver model manual for parents unambiguously suggest that such behaviors should be eliminated if they hinder intervention: “stereotypies (…) can occupy children’s attention so that they are not watching and learning from others. (…) They do not provide the child with any new information ability, so they do not promote learning” ([57] pp 122). The example of a precocious interest in written material is difficult to reconcile with such statements.

Several types of RIRBs have now been distinguished. Some, such as echolalic or hyperlexical behaviors, as well as intense interests in general, are related to information processing and seeking and are highly specific to autism. Our position is that they should be encouraged and nurtured, as they are manifestation of interest in all children. We feel that it is unjustified to try to eliminate manifestations of interest, even if these RIRBs are “obsessive”. To continue our autism-deafness analogy, we would suggest that the Denver model view of stereotypies is the modern equivalent of the repression of sign language commonly practiced in 19th century deaf-and-mute institutions. Others are emotion-related RIRBs, and are manifestations of positive (hand-flapping) or negative (minor self-injury) emotions related to this information. These RIRBs, observed in typical children as well as in multiple neurodevelopmental variants, should be accepted as such. Finally, the “captivity” subgroup of RIRBs (including rocking, or wandering in circles) is not specific to autism. Such behaviors are observed even in animals and are caused by information deprivation. We predict that feeding the first RIRB category, information-seeking behavior, should decrease “captivity” behaviors.

Some repetitive behaviors occupy much of the child’s time, and when interrupted by daily routines, may be associated with tantrums or behaviors dangerous for the child or others (for example, running away). Autistic interests should therefore be included within an educational framework, consistent with safety and educational requirements. However, this does not modify our position: RIRBs that reflect the search for and processing of information relevant to the child’s interest should be encouraged. They represent the motivational foundation on which the education of the autistic child should be based. This is a drastic departure from using them as a pretext for social routine, such as in NDBI.

## What should future studies focus on?

The empirical program resulting from these position statements can be summarized in five testable predictions: (1) a large proportion of prototypical autistic children have normal non-verbal intelligence, despite presenting with a “frank” [17] phenotype, and will develop communicative language later; (2) increasing the exposure of autistic children to non-social language material should favor their long-term linguistic development and (3) decrease their RIRBS and problematic behaviors; (4) lateral tutorship, favoring delayed imitation, should result in better adaptation as adults than the use of EIBI techniques; (5) inclusion in community daycare following these principles should result in greater and more cost-effective improvement of social abilities and child well-being than child-directed, individualized, professionalized, and specialized intervention.

Prediction 1: intellectual disability is associated with syndromic autism, not prototypical autism.

Based on the principles outlined above, we need to rethink the status of intelligence in the diverse children receiving diagnoses of autism. It is frequently overlooked that Kanner discarded intellectual disability in his first group of 11 autistic children, for whom he reported indices of normal intelligence. Actual intellectual disability is rare in non-syndromic autism, but can be confused with it. The early measurement of intelligence in institutionalized adults [33] resulted in most being found to have an intellectual disability when tested with verbally loaded instruments. Even the non-verbal subscales of these instruments can yield deceptive results, as the instructions given are also heavily language-loaded [14]. A certain proportion of non-verbal autistic children may, indeed, have a *false* intellectual disability. Our group has provided substantial evidence that intelligence is “at risk of being underestimated” in young non-verbal autistic individuals [11]. This conclusion is supported by the absence of strong indicators of actual intellectual disability, such as late walking, in most prototypical autistic children. Accordingly, walking age is not related to Mullen-measured intelligence in autistic children, in contrast to findings for children with a non-autistic intellectual disability [4]. Reported rates of intellectual disability in autism vary by approximately 40% between US states providing data (Utah: 20%; South Carolina: 60%; [9]). This suggests that the proportion of autistic individuals with intellectual disability cannot be accurately determined with the instruments currently used for its measurement.

The reported rates of intellectual disability among individuals with an autistic phenotype may result from a combination of measurement error and the deleterious effects of the current definition of autism, which groups non-syndromic and syndromic autism neurodevelopmental disorders together. Identified neurogenetic conditions, as well as other childhood psychiatric and learning disabilities, reach the cut-off for an autism diagnosis due to their negative signs [39], particularly in the social domain. There may be various reasons for not presenting typical social behaviors, which may result in a mistaken diagnosis of autism, due to the absence of exclusion criteria in the current definition.

The presumption of normal, non-verbal intelligence is at odds with the “learning to learn” behaviorist principle that currently dominates early intervention. It suggests that we should instead rely on autistic intelligence, the most frequent eventuality in prototypical autistic children. Future research on autistic intelligence should involve prospective studies of behavioral predictors of intelligence, and on the materials and information most likely to reveal these behaviors [11].

In summary, prototypical autistic children presenting at least one domain of competence clearly above the baseline level, and who are functionally non-verbal between the ages of 2 and 4 years [62], should not present any detectable de novo or inherited copy number variations [28, 54, 56]. They should also begin to walk within the normal age range [4], and have non-verbal intelligence within the normal range at school age [14, 16], despite their strong past or current autistic phenotype.

Prediction 2: favoring access to written verbal material and auditory non-communicative language should favor the long-term development of speech

Speech development is generally delayed in autistic children, who frequently have no speech or speech limited echolalia [33]. In prototypical autism, there is a substantial delay in speech associated with normal non-verbal intelligence and a reorganization of brain allocation and function in favor of perception, particularly visual [58]. This results in autistic toddlers being particularly dependent on visual inputs to obtain the complex information they require. For example, a substantial proportion of nonverbal autistic preschoolers become interested in written material before communicative speech. Extensive reviews of autistic accomplishments [41] have concluded that autistic expertise is based on the detection and manipulation of complex perceptual and formal structures. Relational and emotional components of education may plausibly favor speech development, but are not directly implicated in its mechanisms.

This suggests that autistic children go through an alternative sequence of learning, in which speech development occurs later than in typical children. Exposure to and active involvement in oral language, an essential step in the mastering of speech in non-autistic children, may initially be of little relevance to autistic children. In contrast, subtitles, continuous information on TV screens, YouTube URLs, film credits, magnetic boards, keyboards, and audio tapes of “Dora” programs may be much more pertinent. This does not mean that it is pointless to speak to autistic children, but rather that the capacity to process communicative speech and benefit from it may mature after noncommunicative speech and written language. Similarly, the oral component of speech is minimally relevant for congenitally deaf children born to hearing parents, but may become relevant once language has been learned by other ways.

The fact that autistic children do not *primarily* use oral language in a communicative way has important consequences for intervention. We predict that exposure to written and oral *non*-*communicative* speech will help autistic children to use speech once they develop the ability to map language structure onto oral speech [41]. We also predict that the bonding effect of interactive routines in typical children may have an equivalent in the explicit *provision of materials of this type* to autistic children by their caregivers. A bedtime book-reading routine, repeatedly watching video programs including subtitles with the child, and joint activities (categorizing by font, geometric arrangement, matching, ordering magnetic letters), may be the autistic prerequisites for the future use of speech in a social context. This prediction is based on our systematic review of hyperlexia [49]: the non-communicative use of letters is not a dead-end in the autistic pathway leading to the development of language use.

Prediction 3: including periods of free access to material of “special interest” in the child’s time-schedule will decrease “captivity” RIRBs, and problematic behaviors.

We hypothesize that many of the autistic behaviors considered to be problematic, which most intervention techniques aim to suppress, are actually related to the search for information. In contrast, repression of the behaviors associated with this searching is likely to result in information deprivation in individuals seeking information through unconventional channels, such as autistic and deaf people. This, in turn, would lead to an increase in nonspecific captivity and repetitive behaviors associated with negative emotions.

One practical implication of this hypothesis is that autistic children should be exposed to material of interest, just as non-autistic children are exposed to situations and opportunities that will help them to develop their intelligence. The initial evaluation of repetitive behaviors for diagnostic purposes provides information about the domains in which the child has developed intense, focused interest. This information may be extended and expanded by observing searching behaviors, together with the emotions associated with the provision (flapping) or withdrawal (tantrums, with or without self-injury) of relevant information. The interests of the autistic child depend on the age at which the diagnosis is made. They may initially manifest as a search for a large class of objects with a specific physical characteristic, such as objects that shine, rotate, or can be lined up. Later, restricted interests may be overtly restricted to a specific class of objects (e.g., fire extinguishers, keys, wheels), or to the consistent use of instruments providing access to written numbers and letters, such as screens, computers, tablets, and magnetic letters. Free access to these objects could, therefore, be actively integrated into the child’s daily schedule.

We predict that routinely providing autistic children with their objects of interest will *decrease* behaviors related to both the unsuccessful search for interesting information, and its withdrawal. We predict that knowing when this information will be accessible, through representation of the time window during which objects of interest will be provided on a visual timetable, for example, should alleviate the tantrums associated with its unpredictable withdrawal. This will help the child to understand that although the computer tablet is taken away when it is time for dinner, it will be returned the next day. If the material is sufficiently complex, this should also allow the autistic child to explore and manipulate it in the vicinity of his or her carers, thereby alleviating wandering/captivity behaviors, and favoring the further development of autistic expertise, as well as usable language.

Prediction 4: lateral tutorship of autistic preschoolers in natural parental and daycare settings improves the well-being of parents and school age children more than early, personalized, professional, face-to-face intervention.

The active avoidance of peers is the exception, not the rule, in autism. When left to move freely, except for a minority of escape behaviors, autistic toddlers occupy themselves *among* other people, despite not overtly interacting with them. Autistic children have an obvious preference for parallel play over interactive play. However, parallel play may be the paradigm in autistic social bonding, providing the child with an opportunity to learn from other human beings. If autistic children are allowed to use their own material of interest, they may display delayed imitation of behaviors they have observed [44]. We define “lateral tutorship” as the autistic equivalent of face-to face play interaction of non-autistic children. In lateral tutorship, an adult performs an activity on a material selected from among the child’s interests, such as manipulating a tablet application while the child manipulates, at his side, the same application on his or her own tablet. Lateral tutorship involves actively providing the autistic child with material enriching the dimensions he or she is initially attracted to, and working on that material in parallel with the child. It is based on the observation that autistic toddlers actively reproduce what they have seen within their domain of interest, albeit with a delay. Last, lateral tutorship exposes the child to finalized, completed, and contextualized actions, whereas AIBI learning techniques consist of prompting, shaping, fading, chaining, differential reinforcement discrimination training, and errorless learning of mostly fragmented actions.

We predict that activities carried out in parallel with the child, without the child being addressed directly through gestures or speech, may favor the development of a type of social interaction *based on a common interest* rather than *on a common appearance.* There is no need for the child to master the “prerequisites” of typical face-to-face interaction (smile, overt joint attention, pointing) for this purpose. Instead of focusing on the overt indices of typical socialization, lateral tutorship favors *actual* social bonding with the parents: doing something close to someone else that is protective and cares for you. We predict that lateral tutorship will result in the incidental learning of new manipulations and uses of the material provided. We also predict that studies of the active elements of intervention will show that, in the long term, lateral tutorship results in a level of actual social integration that is at least as good as that achieved with face-to-face, EIBI-type, socially oriented educational interaction or play sessions.

Prediction 5: inclusion in regular, but supervised daycare centers and parental support, associated with on-site crisis solving, is a more cost-effective educational intervention than EIBI/NDBI.

There is no demonstrated dose-dependent effect for the efficacy of EIBI in terms of the number of hours/week or the number of years of therapy. The reported changes are common to different techniques, and reach a plateau within the first year of therapy [12]. This raises questions as to why the practice guidelines of developed countries, except for the UK, continue to recommend, while failing to provide, 20–25 h per week of intervention by autism professionals. This recommendation defines an unreachable and unjustified target that is used secondarily to calculate the alleged social “cost” of autism. We, therefore, strongly recommend including most autistic children in regular daycare centers that receive additional financial and professional on-site support when they accept a certain proportion of children with neurodevelopmental variants, including autism. Over and above the issue of cost, this inclusion in regular settings provides autistic children with information about the real social world and allows them to participate, even if only minimally, in *real* social routines. We predict that the indirect tolerance of non-autistic socialization observed when prototypical autistic children are integrated into regular daycare centers, will later help these children to better integrate into the school environment. Inclusion also provides non-autistic children with an opportunity to see that not everybody is like themselves, with potential societal benefits for the future. Finally, concerning true problematic behaviors, such as intractable tantrums, running away, or ultra-aggressive behaviors, only those that cannot be dealt with by preventive adaptation to the autistic child by on-site professionals, or those exceeding the tolerance levels accepted for other children, justify focused intervention and transient withdrawal of the child.

The balance between the time spent interacting with adults and the time in which the child decides on his own activities under adult supervision should not differ between autistic and non-autistic children. The cliché that an autistic child playing with his object of interest does not learn, confuses restricted interest with no interests at all. The combination of regular daycare integration with family and individual activities, involving material that is interesting to an autistic child, is of similar intensity to an autism-tailored intervention. Concerning methodology, assessment of the true validity and efficiency of a specific intervention should include optimal and inclusive “untreated” comparison groups in non-segregated settings in which “untreated” children are included in high-quality, regular daycare centers, selected on a voluntary basis, rather than less-demanding, therapeutic settings or disorganized private sessions.

We predict that such comparison groups will, in the long term, be shown to result in measurable well-being that is at least as good as that for EIBI-treated groups. The supposed benefit of a 1:1 ratio of autism professionals to children recommended for EIBI should be compared to that of opportunistic access to neurodevelopmental professionals, working in a 1:8 ratio in association with typical childcare professionals in regular daycare centers. The fact that the Denver model is “fun to apply” does not justify its cost and level of “professionalization”, exceeding 30 h/week.

## In conclusion

There is currently no scientific, ethical, or societal justification for EIBI. The degree of improvement in the well-being and adaptive abilities of autistic children and adults does not justify the withdrawal of autistic children from the regular educational system and culture provided by their families and countries. However, recent contestations of the scientific value of EIBI and the magnitude of changes due to this approach, and its rejection by many members of the adult autistic community have not yet had any marked influence on public health policies, intervention targets, or scientific understanding of autism. The aims of autism science are still normative and normocentric, from suppressing autism itself to mimicking non-autistic social behavior. As highlighted by autistic adults, autism is part of the human condition and is here to stay, despite the triumphalism of some scientists. The purpose of educational and child psychiatry interventions should rather be to allow the individual to achieve an abstract level of happiness, personal accomplishment, access to cultural material, and social integration, an essential human right, regardless of the way in which this is achieved and the form that it takes. An acceptation of autistic humanity begins by changing targets, methods, and efficiency variables of the education offered to autistic children, in favor of a strengths-informed education. Science should follow societal evolution, as for other neurodevelopmental variants, now accepted as full members of the human community. Whether our discipline, child psychiatry, supports or slows this movement will be severely judged in the future.
